# Microbial protein targets: towards understanding and intervention

**DOI:** 10.1017/S0031182017002037

**Published:** 2017-11-16

**Authors:** PAUL W. DENNY

**Affiliations:** Department of Biosciences, Durham University, Lower Mountjoy, Stockton Road, Durham DH1 3LE, UK

**Keywords:** Antimicrobial targets, bacteria, pathogens, protozoa

## Abstract

The rise of antimicrobial resistance, coupled with a lack of industrial focus on antimicrobial discovery over preceding decades, has brought the world to a crisis point. With both human and animal health set to decline due to increased disease burdens caused by near untreatable microbial pathogens, there is an urgent need to identify new antimicrobials. Central to this is the elucidation of new, robustly validated, drug targets. Informed by industrial practice and concerns, the use of both biological and chemical tools in validation is key. In parallel, repurposing approved drugs for use as antimicrobials may provide both new treatments and identify new targets, whilst improved understanding of pharmacology will help develop and progress good ‘hits’ with the required rapidity. In recognition of the need to increase research efforts in these areas, in 14–16 September 2017, the British Society for Parasitology (BSP) Autumn Symposium was hosted at Durham University with the title: *Microbial Protein Targets: towards understanding and intervention.* Staged in collaboration with the Royal Society of Chemistry (RSC) Chemistry Biology Interface Division (CBID), the core aim was to bring together leading researchers working across disciplines to imagine novel approaches towards combating infection and antimicrobial resistance. Sessions were held on: ‘Anti-infective discovery, an overview’; ‘Omic approaches to target validation’; ‘Genetic approaches to target validation’; ‘Drug target structure and drug discovery’; ‘Fragment-based approaches to drug discovery’; and ‘Chemical approaches to target validation’. Here, we introduce a series of review and primary research articles from selected contributors to the Symposium, giving an overview of progress in understanding antimicrobial targets and developing new drugs. The Symposium was organized by Paul Denny (Durham) for the BSP and Patrick Steel (Durham) for RSC CBID.

## INTRODUCTION

The threat posed by anti-microbial resistance (AMR) has been well publicized with respect to bacterial pathogens, and the need to identify, validate and exploit new drug targets emphasized (Brown and Wright, 2016). However, similar pressures exist for protozoan pathogens (Tuteja, 2017; Uliana *et al.* 2017). The Special Issue of Parasitology introduced here is focused on global infectious disease, namely the causative agents of the bacterial disease tuberculosis (TB; *Mycobacterium tuberculosis*) and the protozoal infections malaria, toxoplasmosis, leishmaniasis, African Sleeping Sickness and Chagas disease (the apicomplexans *Plasmodium* spp. and *Toxoplasma gondii*; and the kinetoplastids *Leishmania* spp., *Trypanosoma brucei* sp. and *T. cruzi*, respectively). Each of these poses serious challenges to health and wellbeing, and offer a multitude of challenges to effective treatment. Responsible for 1·8 m deaths, and with nearly 5% of the 10 million or more new cases each year showing multi-drug resistance, *M. tuberculosis* remains a serious problem, particularly in low- and middle-income countries (WHO, 2015). Similarly, although incidence levels fell more than 20% during 2010–2015, infection with mosquito-borne *Plasmodium falciparum*, the causative agent of serious malaria, remains a major global health problem leading to more than 200 m new cases and 400 000 deaths/year (WHO, 2016*a*). The related apicomplexan protozoa *Toxoplasma,* classified by the Centers for Disease Control as causing a Neglected Parasitic Disease, chronically infects 30–60 m people in the USA alone, where it is considered a major food-borne pathogen (CDC, 2017). The kinetoplastid parasites *Leishmania* spp., *T. cruzi* and *T. brucei* sp. are insect-borne causes of Neglected Tropical Diseases (NTDs). However, whilst cases for African Sleeping Sickness caused by the later are declining [<3000 in 2015; (WHO, 2016*b*)], there are up to 1 m new cases of leishmaniasis per year, leading to 20 000 deaths and 6–7 m people remain infected with the parasite that causes Chagas disease (WHO, 2017). Indeed, the battle against *Leishmania* spp. and *T. cruzi* infection has recently been described as a losing one (Hotez and Aksoy, 2017).

For the global infections outlined above, the available drugs have limitations of efficacy, tolerance and/or administration, and cases of AMR are emerging or rampant. To address these problems, for both bacterial and protozoal pathogens there is a well recognized need to identify new targets for antimicrobial intervention (Brown and Wright, 2016; Muller and Hemphill, 2016). However, the identification, validation and understanding of new protein targets is not a straightforward process. For example, within the pharmaceutical industry there are wide-spread concerns regarding the reproducibility of drug target validation studies across a range of disease states (Jones, 2016). Against this backdrop, the British Society for Parasitology 2016 Autumn Symposium focused on the identification, understanding and exploitation of targets for antibacterial and anti-protozoal intervention. In recognition of the industry concerns outlined above, the focus on cross-disciplinary analyses of putative targets was designed to answer the call to ‘*embrace chemistry at the interface with biology’* (Jones, 2016) and provide more robustly triaged drug targets. This approach necessitates the application of both state-of-the-art genetic and chemical tools to answer key questions in bioscience and robustly validate new drug targets in both bacterial and protozoan pathogens.

## SEARCH FOR ANTIMICROBIAL TARGETS

With a crisis in antimicrobial resistance upon us, and the persistence of neglected infectious diseases (e.g. NTDs), new drug leads need to be rapidly identified. High-throughput screening (HTS) remains at the centre of drug discovery and can be carried out using either *in vitro* assays against validated targets or phenotypic assays against the pathogen itself (Denny and Steel, 2014; Norcliffe *et al.* 2014). Recent high content phenotypic screening across the kinetoplastids gave a disappointingly low number of novel potent hits against *Leishmania donovani* when compared with the related parasite *T. brucei* (Pena *et al.* 2015). Phenotypic HTS has been successfully carried out against *M. tuberculosis,* for example using genetically modified bacteria in a resistance based screen (Cox *et al.* 2016). However, as for the kinetoplastids this has proven problematic, due in large part to the slow growth rate of *M. tuberculosis* (White *et al.* 2016). These studies demonstrated that target-based screening remains vital of for antimicrobial discovery and, of course, this relies upon the provision of high quality, fully validated, antimicrobial drug targets.

Natural product antibacterials (antibiotics) targeting the cell wall have long been in clinical use, many of these are directed against peptidoglycan, the principle component of Gram-positive and -negative bacterial walls (Muller *et al.* 2017). Such antimicrobials show excellent selectivity for the synthesis of this non-mammalian structure; however, they are at the forefront of concerns regarding AMR. Likewise, several anti-TB agents target cell wall synthesis, however the *M. tuberculosis* wall has several unique features, which present challenges for the development new chemotherapeutics. For example, the long-chain mycolic acids which cover the cell surface facilitate the intercalation of acyl lipids forming a waxy outer membrane, which forms a hydrophobic barrier. Despite these obstacles the *M. tuberculosis* wall and its biosynthesis remains an important and attractive target for novel anti-TB drugs, as concluded in the first review in this Special Issue (Abrahams and Besra, 2016). Abrahams and Besra present the biosynthesis of this essential structural and permeability barrier as being the ‘Achilles heel’ of this pathogen and open up the prospect of modern approaches to drug discovery (e.g. HTS) identifying novel therapeutics.

Similarly, the protozoan sphingolipid biosynthetic pathway has been proposed as a possible drug target for kinetoplastid (e.g. *Leishmania* spp. and *T. brucei*) and apicomplexan (*Plasmodium* spp. and *Toxoplasma*) eukaryotic pathogens (Mina *et al.* 2010, 2011; Young *et al.* 2012; Coppens, 2013; Pratt *et al.* 2013). Against the backdrop of ancient, toxic therapies and rising AMR (Barrett and Croft, 2014) the essentiality of sphingolipids, and the potential to target their biosynthesis, has seen growing interest. In a ‘state-of-the-art’ review in this Special Issue, Mina and Denny discuss possibilities and pitfalls of targeting this biosynthetic pathway, considering both parasite *de novo* synthesis and host scavenging (Mina and Denny, 2017). Key differences between the mammalian host sphingolipid biosynthetic pathway and that of both kinetoplastid (Denny *et al.* 2006; Zhang *et al.* 2010) and apicomplexan (Coppens, 2013; Pratt *et al.* 2013; Mina and Denny, 2017) protozoan parasites, have fuelled this endeavour. In a companion piece, Alqaisi *et al.* (2017) describe an investigation of the mode of action of a reported inhibitor of *Toxoplasma* sphingolipid biosynthesis, aureobasidin A. However, whilst this natural compound is antiparasitic against both acute and chronic forms, parasite sphingolipid biosynthesis was unaffected.

Remaining in the field of lipid biochemistry, protein acylation has long been proposed as a target of novel antiprotozoals, with the enzyme responsible for the essential *N-*myristoylation of proteins [*N*-myristoyl transferase (NMT)] identified as a potential drug target in apicomplexan (Gunaratne *et al.* 2000) and kinetoplastid (Price *et al.* 2003) protozoan parasites. In this Issue, the use of chemical proteomic approaches to analyse and validate such post-translation modifications is discussed, with reference to both *N-*myristoylation and *S*-palmitoylation (Ritzefeld *et al.* 2017). The use of chemical tools (such as acyl biotin exchange and metabolic tagging with click chemistry) is essential to fully understand the downstream effects of enzyme inhibition and provide further validation of targets and inhibitors (Tate *et al.* 2014; Ritzefeld *et al.* 2017).

A unique target in kinetoplastid protozoa is the mitochondrial protein, trypanosome alternative oxidase (TAO). This target is now well characterized in *T. brucei* where it has ubiquinol oxidase activity and is expressed more than 100-fold more in pathogenic bloodstream forms (Chaudhuri *et al.* 1998). Functionally it is thought to protect the parasite from oxidative damage (Fang and Beattie, 2003). As reviewed in this issue, inhibitors of TAO have been identified which are able to clear infection *in vivo* (Menzies *et al.* 2016).

Collectively, these studies and associated reviews emphasize the place for a target-directed approach in antimicrobial discover and emphasize the importance of chemical approaches for the understanding and validation of drug targets.

## EXPLORATION AND EXPLOITATION OF ANTIMICROBIAL TARGETS

As discussed above, screening of phenotypic changes in response to chemical assault is one approach to identifying new leads as antiparasitics, although obtaining informative readouts from such assays can be complex (Denny and Steel, 2014; Norcliffe *et al.* 2014). In this Special Issue, the use of *in silico* synchronization, using defined cell parameters, to more readily analyse the cell cycle of *T. brucei* is proposed (Morriswood and Engstler, 2017). Such an automated process could increase throughput and standardize data quantitation, perhaps providing more robust phenotypic data. However, if an antimicrobial target is in hand, the search for inhibitors for use as drug leads or chemical tools for further validation and understanding, can employ conventional HTS. Typically, this involves screening a large, diverse compound library (>100 k) against a protein target in a multiwell formatted biochemical assay (Denny and Steel, 2014; Norcliffe *et al.* 2014). However, fragment-based approaches are now often run alongside such HTS, and have been the key to success in several drug discovery programmes (Congreve *et al.* 2008; Scott *et al.* 2012). The application of this approach in the discovery of inhibitors of *M. tuberculosis* targets is reviewed here by Marchetti *et al.* A fragment-based approach involves screening a small library (1000–5000) of fragments (<250 Da) against a protein target, and identifying weak binders by using a variety of biophysical tools such as surface plasmon resonance and nuclear magnetic resonance (Marchetti *et al.* 2016). Notable successes include the identification, using thermal shift assays, of fragments binding to EthR, a TetR-type transcriptional repressor that underlies *M. tuberculosis* resistance to the second-line drug ethionamide (Villemagne *et al.* 2014). Following analyses of an X-ray co-crystal structure, a virtual library was designed and screened *in silico*, leading to the identification of derivatives with high *in vitro* activity (Tatum *et al.* 2013; Villemagne *et al.* 2014). This demonstrates the centrality of high resolution protein structures to fragment-based ligand-discovery approaches (Murray and Blundell, 2010), and the potential of *in silico* screening.

Such structure-based approaches can be considered as applicable to all protein targets, in this Issue the calcium-dependent protein kinases (CDPK) from *Toxoplasma* are considered as targets for such an approach (Cardew *et al.* 2017). CDPK are restricted to plants and protozoa and have been genetically demonstrated to be essential in multiple systems, including *Toxoplasma,* presenting them as attractive drug targets (Long *et al.* 2016; Wang *et al.* 2016). Structure-based approaches have led to the discovery of potent CDPK inhibitors, with specificity with respect to mammalian kinases and good antiparasitic activity (Lourido *et al.* 2013; Zhang *et al.* 2014; Moine *et al.* 2015).

This work, taken together, illustrates the power of utilising diverse chemical and biophysical approaches to identify novel inhibitors and antimicrobial lead compounds.

## ALTERNATIVE APPROACHES AND DOWNSTREAM NECESSITY

Whilst target-based approaches to antimicrobial discovery remain central, the exceedingly long history of repurposing drugs for use as antiparasitics demonstrates that we should not be too narrow in our thinking. As reviewed in this Special Issue repurposing has the potential to significantly reduce the costs of antimicrobial discovery by bypassing the initial development phases necessary for new chemical entities (Charlton *et al.* 2017). Charlton *et al*. discuss the current prominence of such drugs in the treatment of leishmaniasis, for example amphotericin B, which was developed as an antifungal but is currently in use in the South Asian visceral leishmaniais elimination programme (Gurunath *et al.* 2014). In addition, they review the history and potential of other drugs developed as, for example, antiviral and anticancer agents, for use as antileishmanials. However, as Charlton et al. recognize, whilst the discovery and repurposing of existing pharmaceuticals will save time and money in the vital search for safe, effective and affordable antileishmanials, the identification of the mode of action of such drugs is essential of further development (Ritzefeld *et al.* 2017).

The discovery and validation of antimicrobial targets and potent inhibitors is, of course, a vital component of a drug discovery programme. However, these *in vitro* approaches provide no indication as to the ability of identified chemical entities to reach the target pathogen within the host. The particularly acute challenges of this stage in the discovery pipeline for pathogens sequestered within host cells, such as *Leishmania* spp. and *M. tuberculosis,* is reviewed in this Issue (Croft, 2017). The integration of pharmacokinetics (PK), pharmacodynamics (PD) and physiological-modelling into the antimicrobial drug discovery process has been previously reviewed (Edginton *et al.* 2008; Nielsen and Friberg, 2013), and the importance of PK–PD analyses in *M. tuberculosis* drug design demonstrated (Davies and Nuermberger, 2008; Dartois, 2014). Given that *Leishmania* spp. occupy a similar intracellular site to *M. tuberculosis,* Croft considers the application of these approaches to antileishmanial discovery, concluding that uniform approaches at all levels of the pipeline are vital to ensure the development process can proceed as rapidly as possible.

### Concluding remarks

The collection within this Special Issue illustrates the centrality of high quality target validation (using both biological and physical methodologies); the importance of multifaceted inhibitor discovery (integrating HTS and biophysical approaches); and the requirement to consider physiological factors (such as PK–PD) in antimicrobial discovery. In summary, the adoption of the multidisciplinary approaches outlined is essential to accelerate the discovery of new drugs to treat the most prevalent, and often intractable, global infections caused by both bacterial and protozoal pathogens.
